# A Novel Surface Plasmon Resonance Imaging (SPRi) Biosensor for the Determination of Bovine Interleukin-10: Development, Validation, and Application in Biological Fluids

**DOI:** 10.3390/ijms262110395

**Published:** 2025-10-25

**Authors:** Aleksandra Pytel, Dawid Tobolski, Piotr Skup, Justyna Gargaś, Sylwia Flis, Zdzisław Gajewski, Ewa Gorodkiewicz, Krzysztof Papis

**Affiliations:** 1 Department of Large Animal Diseases and Clinic, Institute of Veterinary Medicine, Warsaw University of Life Sciences, 02-776 Warsaw, Poland; 2 Center of Translational Medicine, Warsaw University of Life Sciences, 02-797 Warsaw, Poland; sylwia_flis@sggw.edu.pl (S.F.); zdzislaw_gajewski@sggw.edu.pl (Z.G.); krzysztof_papis@sggw.edu.pl (K.P.); 3 Bovisvet Veterinary Practice of Reproduction and Cattle Diseases, 08-307 Kosierady Wielkie, Poland; piotr@bovisvet.com; 4 NeuroRepair Department, Mossakowski Medical Research Institute, Polish Academy of Sciences, 02-106 Warsaw, Poland; jgargas@imdik.pan.pl; 5 Bioanalysis Laboratory, Faculty of Chemistry, University of Bialystok, 15-245 Bialystok, Poland; ewka@uwb.edu.pl; 6 nOvum Fertility Clinic, 02-807 Warsaw, Poland

**Keywords:** interleukin-10, SPRi biosensor, bovine, follicular fluid, serum, immunoassay, veterinary diagnostics

## Abstract

Interleukin-10 (IL-10) is a pleiotropic cytokine that is pivotal in regulating the immune response. Its involvement in the pathophysiology of bovine diseases and its potential influence on oocyte developmental competence make it an important target for diagnostics and research. This study aimed to develop and validate a novel, rapid, and sensitive analytical tool for its quantification. A specific biosensor based on Surface Plasmon Resonance Imaging (SPRi) was developed for the precise quantification of bovine IL-10, utilizing a polyclonal rabbit antibody immobilized on a gold chip for direct capture from complex biological matrices. The method was validated for its analytical performance, including linearity, sensitivity, precision, and selectivity. The developed method is characterized by a wide diagnostic range (1–1000 pg/mL) and high sensitivity, with a limit of detection (LOD) of 0.45 pg/mL and a limit of quantification (LOQ) of 1.49 pg/mL. The biosensor was successfully applied to measure IL-10 concentrations in bovine serum and follicular fluid, revealing significantly higher levels in follicular fluid. The validated SPRi biosensor is a rapid, sensitive, and cost-effective tool for determining IL-10 levels. Its successful application confirms its utility for veterinary diagnostics and highlights its potential for research in reproductive biology, particularly for assessing the follicular microenvironment.

## 1. Introduction

Interleukin-10 (IL-10) is a cytokine of fundamental importance for maintaining immune homeostasis, acting as a key regulator of innate and adaptive immune responses. Its primary function involves the suppression of pro-inflammatory cytokine production, such as tumor necrosis factor-alpha (TNF-α), interleukin-1β (IL-1β), and interleukin-6 (IL-6), as well as the limitation of antigen-presenting cell (APC) activation. This mechanism prevents excessive and potentially tissue-damaging inflammatory reactions [1]. In veterinary medicine, the role of IL-10 is particularly significant in the context of inflammatory diseases in cattle. During mastitis, IL-10 functions as an anti-inflammatory factor, although its concentration can vary depending on the etiological determinants and the stage of infection [2,3]. In the case of paratuberculosis, a chronic disease caused by Mycobacterium avium subsp. paratuberculosis (MAP), elevated IL-10 levels can paradoxically promote pathogen survival within the host by dampening the effective immune response [4].

Beyond its role in pathology, a growing body of evidence highlights the critical importance of cytokines, including IL-10, in reproductive physiology. The proper development of the ovarian follicle and the oocyte’s acquisition of complete developmental competence depend on complex interactions within the follicular microenvironment. IL-10, produced locally by granulosa cells and resident immune cells, can modulate this environment, influencing oocyte quality and maturation processes [5,6]. Consequently, the concentration of IL-10 in follicular fluid is emerging as a potential biomarker that could be utilized to assess gamete quality in assisted reproductive technology (ART) programs, including in vitro embryo production (IVEP) [7].

Standard methods for measuring IL-10, such as enzyme-linked immunosorbent assay (ELISA) and multiplex bead-based assays, suffer from notable limitations despite their widespread use. These methods are labor-intensive, involve multiple incubation and washing steps, require costly reagents (enzyme-labeled antibodies), and need relatively large sample volumes. Moreover, their sensitivity may not be adequate for detecting low cytokine levels, resulting in values below the detection limit [8]. Studies in murine models have also indicated that IL-10 levels measured by immunoassays might not accurately reflect the cytokine’s biological activity, complicating the interpretation of results [9]. A similar issue with IL-10 detection in bovine follicular fluid was reported by Alrabiah et al. (2021), who found IL-10 in only three of twelve samples; the levels in the other samples were below the detection limit [10].

In response to these challenges, this study aimed to develop a new analytical method based on Surface Plasmon Resonance Imaging (SPRi). SPRi biosensors offer an attractive alternative, as they enable label-free, real-time analysis, are characterized by high sensitivity, require minimal sample volumes, and allow for the simultaneous analysis of multiple samples on a single chip [11,12]. This paper presents the complete procedure for the construction, optimization, and validation of an SPRi biosensor designed to determine bovine IL-10 and demonstrates its practical application for the analysis of serum and follicular fluid samples.

## 2. Results

### 2.1. Optimization of Assay Conditions

**Figure 1:**
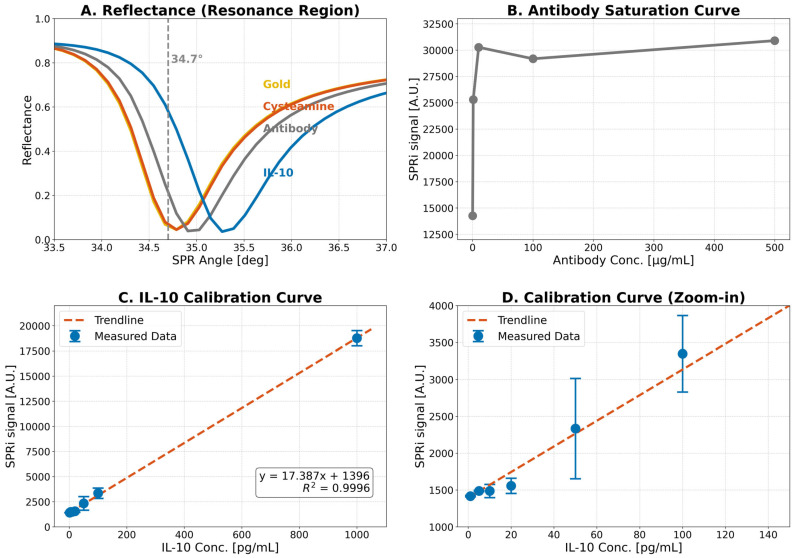
Optimization and calibration of the SPRi biosensor. (**A**) Reflectance curves in the resonance region, showing the SPR dip shift upon sequential layer formation. The selected measurement angle (34.7°) is indicated by the dashed line. (**B**) Antibody saturation curve acquired at pH 7.4, used to determine the optimal antibody immobilization concentration. (**C**) Full calibration curve for IL-10 determination (1–1000 pg/mL) with linear regression fit (R^2^ = 0.9996). (**D**) Magnified view of the low-concentration range of the calibration curve.

The development of the biosensor began with a systematic optimization of key operational parameters, as illustrated in Figure 1. Successful stepwise modification of the sensor surface was confirmed by analyzing reflectance curves in the resonance region (Figure 1A). The curves in Figure 1A show a characteristic shift in the SPR dip upon sequential addition of the cysteamine linker, the antibody, and the IL-10 analyte. Analysis of the resonance region identified an optimal measurement angle of 34.7°, where sensitivity was maximal.

The optimal antibody concentration for immobilization was determined to ensure efficient capture of the analyte. The antibody saturation curve (Figure 1B) demonstrated that the SPRi signal plateaued at a concentration of 10 µg/mL, which was selected for subsequent experiments.

Following optimization, the analytical performance was characterized. A calibration curve was constructed by plotting the mean SPRi signal against IL-10 concentrations ranging from 1 to 1000 pg/mL. The resulting calibration curve exhibited excellent linearity (R^2^ = 0.9996) over a wide diagnostic range (Figure 1C). The method’s reliability for detecting low levels of IL-10 was further confirmed by a magnified view of the low-concentration portion of the calibration curve (Figure 1D). Key analytical parameters were derived based on the complete calibration data. The calculated limit of detection (LOD) was 0.45 pg/mL, and the limit of quantification (LOQ) was 1.49 pg/mL demonstrating the high sensitivity of the method.

### 2.2. Precision, Accuracy, and Repeatability

**Figure 2:**
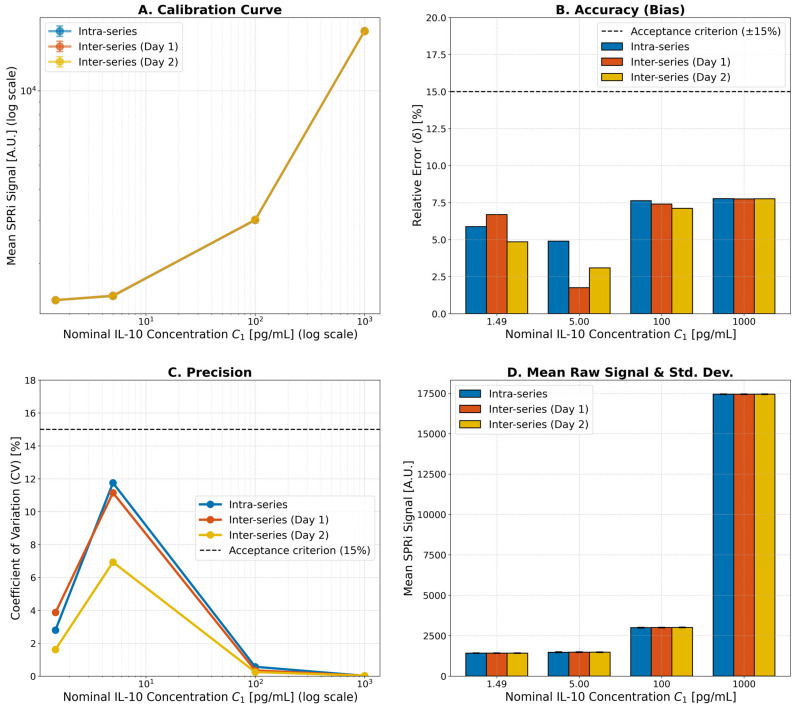
Validation of the biosensor’s analytical performance. (**A**) Calibration curve for IL-10 on a log-log scale. (**B**) Accuracy of the method, showing the relative error (bias) for intra- and inter-series measurements at four IL-10 concentrations. The dashed lines indicate the acceptance criterion of ±15%. (**C**) Precision of the method, showing the coefficient of variation (CV) for the same measurement series. The dashed line indicates the acceptance criterion of 15%. (**D**) Mean raw SPRi signals and standard deviations for the tested concentration levels.

The precision and accuracy of the method were thoroughly evaluated, with the results summarized in Figure 2. The concentrations for the validation samples were calculated based on the mean SPRi signals from the calibration curve, which is presented on a log–log scale (Figure 2A). The accuracy of the method, expressed as the relative error (bias, δ), was assessed at four concentration levels (1.49, 5.00, 100, and 1000 pg/mL) for both intra-series and inter-series (Day 1 and Day 2) measurements. As shown in Figure 2B, the relative error for all tested concentrations remained within the established acceptance criterion of ±15%. Similarly, the precision, expressed as the coefficient of variation (CV), was evaluated for the same measurement series. The results, depicted in Figure 2C, demonstrate that the CV for all levels was also below the 15% acceptance limit. The underlying raw SPRi signals and their standard deviations for each concentration level are shown in Figure 2D, demonstrating measurement consistency. Collectively, these data confirm the high accuracy and precision of the developed method.

### 2.3. Repeatability

A dedicated repeatability test was performed to assess measurement stability and consistency. This involved conducting six consecutive measurements on two different samples: a 500 pg/mL IL-10 standard solution and a representative follicular fluid sample. The results demonstrated excellent repeatability for the method. The calculated coefficient of variation (CV) for the standard solution was exceptionally low at 0.39%. For the more complex biological matrix of follicular fluid, the CV was also very low, at 1.77%. These results indicate that the biosensor provides highly stable and consistent readings over multiple measurements, which is a critical attribute for a reliable analytical method.

### 2.4. Selectivity

The selectivity of the biosensor was confirmed by testing its response to high concentrations (500 and 2000 pg/mL) of structurally related cytokines: IL-1β, IL-6, and IFN-γ. In all cases, the signals generated by these interfering substances were negligible and corresponded to apparent IL-10 concentrations below the method’s LOQ (Table 1). This result unequivocally demonstrates that the immobilized antibody is highly specific to bovine IL-10.

### 2.5. Application to Real Samples

The validated biosensor was successfully applied to quantify IL-10 in a panel of bovine serum and follicular fluid samples. The measured concentrations ranged from 7.08 to 338.25 pg/mL in serum and 320.87 to 810.62 pg/mL in follicular fluid. It is noteworthy that parallel attempts to quantify IL-10 in the same samples using two commercially available ELISA kits (Bovine Interleukin-10 (IL-10) ELISA Kit, MyBioSource, cat. no. MBS8726; and Bovine Interleukin-10 (IL-10) ELISA Kit, Cusabio, cat. no. CSB-E06754Bo) were unsuccessful. In all cases, the concentrations were below the stated limits of quantification for these commercial assays, highlighting the superior sensitivity of the developed SPRi biosensor in analyzing these biological samples. To verify the presence of IL-10 despite these undetectable levels in ELISA assays, dot blot analysis confirmed the presence of IL-10 in the analyzed samples (Table 2).

## 3. Discussion

The present study reports the development and validation of the SPRi biosensor for the quantification of bovine IL-10. Compared with traditional SPR, SPRi provides higher throughput and spatially resolved readouts that facilitate the analysis of molecular interactions [11,13]. In the present bovine context, the sensor addresses a persistent gap in cattle diagnostics, where sensitive and well-validated assays remain limited. Selectivity tests of our new sensor confirmed that signals were specific to IL-10. The platform delivered a diagnostic range of 1–1000 pg/mL, which reduces the need for large dilutions and lowers the risk of measurements falling below the LOD. Taken together, high throughput, spatial resolution, confirmed selectivity, regeneration and broad range position the platform within the wider field of high-sensitivity cytokine analytics [14].

The analytical performance was the principal strength of the biosensor. The LOQ was 1.49 pg/mL, comparable to or superior to many bovine ELISA kits and competitive with other optimized immunoassays. Within the rapidly evolving field of ultrasensitive biosensors, localized surface plasmon resonance (LSPR) platforms with gold-nanoparticle enhancement and electrochemical immunosensors have achieved sub-pg/mL detection for human IL-10 (LOD of 0.33 attograms per milliliter [ag/mL]), yet these formats remain largely research-level and are not available as turnkey kits for veterinary use [15,16,17,18]. Operationally, the SPRi workflow uses ~3 µL per spot, completes nine determinations in ~30 min (conventional ELISAs typically require several hours (≈3–4.5 h) from start to readout [12,19,20]), and allows chip regeneration, providing a low-volume, rapid alternative to ELISAs that typically require ≥50–100 µL per well and to multiplex platforms that demand dedicated readers and extensive matrix optimization [11,21]. Comparable sensitivity has not been validated in cattle for these human-focused platforms, underscoring the importance of the present SPRi approach in bridging the translational gap.

Most ultrasensitive IL-10 biosensing has been developed and validated in non-cattle species. In humans, nanoparticle-assisted LSPR quantified IL-10 in serum on roll-to-roll nanoimprinted substrates, and label-free LSPR enabled approximately 30 min serum measurements in oncology cohorts [15,22]. Electrochemical approaches likewise focus on human matrices: impedimetric sensors in saliva using copper-free click immobilization, ion-sensitive field-effect transistor (ISFET), and electrochemical impedance spectroscopy (EIS) platforms with immunomagnetic preconcentration in saliva or related clinical media, and ZnO-enhanced multiplex impedance devices calibrated in serum and urine [23,24,25]. Evidence in ruminants is limited but relevant: an electrochemiluminescent multiplex assay detected IL-10 in bronchoalveolar lavage fluid from bovine calves, a vertically aligned carbon-nanotube immunosensor has been proposed for bovine paratuberculosis screening, and bead-based multiplex assays have quantified bovine IL-10 in plasma and cell-culture supernatants [26,27,28]. To our knowledge, no bovine study has applied SPRi or any comparably advanced, label-free plasmonic platform to quantify IL-10 directly in native bovine follicular fluid and serum. The present work, therefore, provides the first demonstration of SPRi for bovine IL-10 using microliter inputs, thereby helping bridge the gap between human-focused, ultrasensitive platforms and validated assays for cattle.

The analysis of real biological samples yielded method-relevant insights. Serum IL-10 concentrations measured by SPRi in our bovine cohort ranged from 7.1 to 338.3 pg/mL, whereas FF concentrations were higher (320.9–810.6 pg/mL). These values fall squarely within, or below, the quantitative windows used by bovine ELISAs (typically 2–1000 pg/mL for humane assays), but our FF concentrations are substantially higher than milk measurements reported in bovine mastitis cohorts, emphasizing that matrix and pre-analytics critically determine the measurable signal [3,29,30]. To place these findings into a matrix-by-matrix context, we begin with milk and then consider uterine, systemic, ovarian, and airway compartments.

For milk, bovine IL-10 FF concentrations in our cohort, which were in hundreds of pg/mL, exceeded milk IL-10 means reported by ELISA in bovine subclinical and clinical mastitis. In a large bovine clinical mastitis study, milk cytokines, including IL-10, were quantified in whey prepared by high-speed defatting and reported in activity units (U/mL), which prevents direct conversion to mass concentration and complicates cross-study comparisons [31]. By contrast, bovine subclinical mastitis studies that reported IL-10 in pg/mL consistently yielded low milk concentrations near the lower end of kit standards, for example, approximately 43.5 pg/mL with a 15.6–1000 pg/mL calibration range, values that are an order of magnitude below our FF range and generally similar to or lower than our serum values [3,29]. These matrix effects in milk align with observations from the uterus, where pre-analytics and dilution strongly influence apparent concentrations.

In the uterine compartment, late postpartum cows have been assayed for IL-10 in serum and in uterine washings using single-plex ELISA, with reported intra-assay and inter-assay CV of 10–12% [32,33]. Compared with lavage-based approaches, which typically instill approximately 50 mL PBS with about 40 mL recovery, our SPRi analysis of undiluted compartmental fluid, namely individual FF, avoids an unknown in situ dilution factor inherent to washings, which likely contributes to the higher absolute values observed in FF relative to lavage samples [32,33]. Systemic measurements in blood further contextualize expected circulating levels in cattle.

In blood, serum, or plasma, IL-10 has been measured using single-plex ELISA in cows across several systemic contexts, including the prediction of postpartum reproductive disorders, paratuberculosis status, and clinical or subclinical endometritis windows [30,34,35]. Our bovine serum range of 7.1–338.3 pg/mL overlaps the lower to mid portions of these ELISA calibration spans, often 2–1000 pg/mL, which is consistent with systemic measurements outside strong inflammatory surges and indicates that assayable IL-10 can remain modest in circulation at time points relevant to assisted reproduction [30,34,35]. Early-infection work in bovine foot-and-mouth disease reported IL-10 as optical density units rather than pg/mL, illustrating how reporting format can hinder quantitative benchmarking against our SPRi data [36]. Moving from systemic to ovarian measurements clarifies why FF values are robustly detectable with SPRi.

In the ovarian follicular compartment, peri-ovulatory bovine FF IL-10 measured on a 15-plex Luminex panel at a dilution of 1:100 and normalized to total protein was at or below the LLOQ in most aspirates, whereas our SPRi directly quantified FF IL-10 in the hundreds of pg/mL [37]. Slide-based antibody arrays, such as Quantibody, have been demonstrated largely in non-bovine plasma and FF and typically require dilutions of 1:20 in plasma and 1:100 in FF together with dedicated scanners. These pre-analytical demands, alongside protein normalization, can down-scale apparent concentrations relative to SPRi measurements obtained in minimally diluted samples [38]. The same pattern is evident in another low-protein bovine matrix, the airway.

In the respiratory tract, bovine calves assayed with an electrochemiluminescent multiplex, MSD U-PLEX, exhibited a 24-h rise in IL-10 in bronchoalveolar lavage fluid (BALF) after an aerosolized bacterial lysate, although many cytokines remained below the LLOQ [26]. This example highlights the importance of platform choice and pre-analytics, including lavage recovery and protein normalization, in governing detectability in low-protein matrices. In contrast, our bovine SPRi approach directly quantifies IL-10 in native biofluids without requiring enzymatic amplification or mandatory dilution steps. Taken together, these compartment-specific comparisons provide a coherent framework for interpreting inter-study variability and for positioning our platform in relation to existing bovine assays.

Across bovine milk, uterine washings, blood, FF, and BALF, single-plex ELISAs and multiplex platforms differ in unit systems, calibration spans, required sample volumes, and pre-analytics, and these factors jointly determine the reported IL-10 values [3,26,29,30,31,32,33,34,35,36,37,38]. In this context, the bovine SPRi biosensor provides label-free, single-digit-pg/mL quantitation in microliter volumes and enables direct measurement in scarce matrices such as individual FF, yielding concentrations that align with, yet often exceed, values reported for milk and lavage-based matrices where processing and dilution reduce apparent levels. Although advanced human-focused biosensors exist, veterinary-grade implementations for cattle are not currently available at present, which positions the bovine SPRi platform as a first-in-species analytical capability. This perspective motivates a clear statement of study constraints and the steps required for clinical translation.

Certain limitations warrant consideration. The number of animals was modest, so rigorous validation will require a larger and more heterogeneous cohort that spans relevant physiological and pathological states. Methodologically, side-by-side testing of matched bovine samples by SPRi and at least one orthogonal immunoassay across serum, FF, milk or whey, and uterine washings, with explicit documentation of dilution factors, recovery volumes, unit systems, and normalization strategies, will enable matrix-dependent calibration transfer functions and estimation of proportional or constant bias between platforms [3,30,31,32,33,37,38]. Framing validation in this way helps prioritize the most informative experiments.

A practical next step is a prospective study that links bovine FF IL-10 measured by SPRi to IVEP endpoints, including cleavage and blastocyst rates [5,6,7,8,39]. From an analytical standpoint, adding a small cross-validated cytokine panel, for example IL-6 and IL-10 quantified by a bead-based or slide-array platform, on the same bovine samples will test whether multi-analyte signatures outperform single-analyte readouts and will provide the bridges needed to interpret SPRi values against historical ELISA and multiplex datasets [37,38]. In combination, these studies would integrate first-in-species SPRi measurements with established immunochemical modalities, thereby accelerating the transition from analytical validation to biologically and clinically meaningful endpoints.

## 4. Materials and Methods

### 4.1. Reagents and Materials

Rabbit polyclonal antibody for bovine interleukin-10 (IL-10) was purchased from MyBioSource, Inc. (San Diego, CA, USA). Cysteamine hydrochloride, N-(3-dimethylaminopropyl)-N′-ethylcarbodiimide hydrochloride (EDC), and N-hydroxysuccinimide (NHS) were obtained from Sigma-Aldrich Chemie GmbH (Merck KGaA, Taufkirchen, Germany). Absolute ethanol and other analytical-grade reagents (acetic acid, hydrochloric acid, sodium hydroxide, sodium chloride, sodium carbonate, and sodium acetate) were supplied by Avantor Performance Materials Poland S.A. (formerly POCh S.A., Gliwice, Poland). HBS-ES buffer (pH 7.4; 0.01 M HEPES, 0.15 M NaCl, 0.005% Tween 20, 3 mM EDTA), phosphate-buffered saline (PBS; pH 7.4), and carbonate buffer (pH 8.5) were from Biomed-Lublin Wytwórnia Surowic i Szczepionek S.A. (Lublin, Poland). Argon gas, purity grade 5.0 (≥99.999%) was purchased from Air Liquide Polska Sp. z o.o. (Kraków, Poland).

### 4.2. Biosensor Platform and Instrumentation

**Figure 3:**
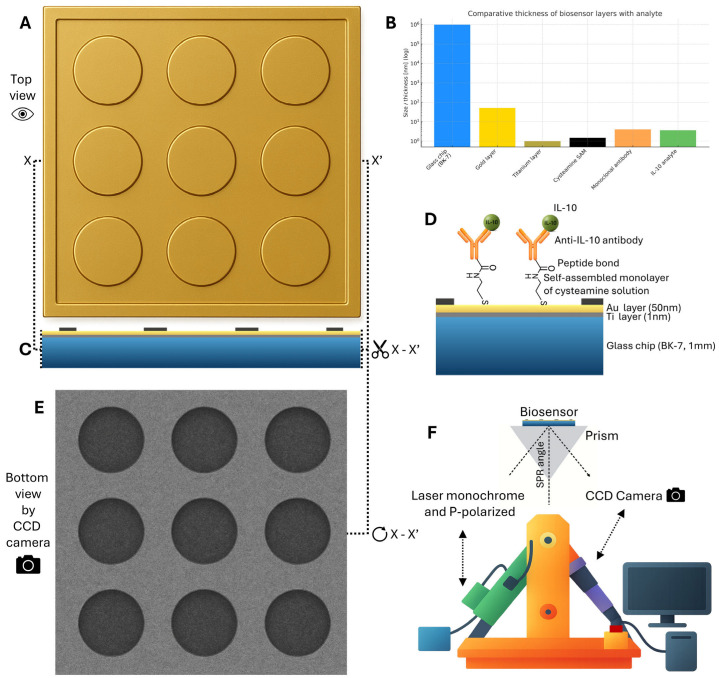
Schematic representation of the SPRi biosensor chip and measurement principle. (**A**) Top-down view of the 3 × 3 multi-array sensor chip. (**B**) Comparative thickness of the biosensor layers, illustrating the scale from the glass substrate to the captured analyte. (**C**) Cross-sectional view of the chip structure along the X-X’ axis. (**D**) Detailed molecular architecture of the sensor surface, showing the covalent immobilization of the anti-IL-10 antibody via a cysteamine self-assembled monolayer (SAM) and the subsequent specific capture of the IL-10 analyte. (**E**) Representative image from the CCD camera, showing the SPR signal as dark spots corresponding to the measurement fields. (**F**) Diagram of the stationary SPRi instrument setup, illustrating the path of the p-polarized laser through the prism to the biosensor surface and the detection of the reflected light by the CCD camera.

The biosensor platform utilized BK-7 glass chips (24 × 24 mm, 1 mm thick) coated with a 1 nm titanium adhesion layer and a 50 nm gold layer (SensEye chips, Ssens B.V., Enschede, The Netherlands). The chip features a 3 × 3 array of measurement sites, enabling nine simultaneous analyses (Figure 3A–E). The gold surface was patterned with a hydrophobic polymer layer to physically separate these sites. The layered structure of the chip is shown in a cross-sectional view in Figure 3C, with a comparative scale of each layer displayed in Figure 3B.

Surface plasmon resonance imaging (SPRi) measurements were carried out on a stationary, custom-built instrument described previously [40,41] (Figure 3F). The optical path comprised a p-polarized 635 nm laser diode, a collimator, a beam expander, a polarizer, a TC-series bi-telecentric lens (Opto Engineering, Mantova, Italy), and a 1.4 MP monochrome CCD camera that captured the reflected beam. Image processing and SPRi signal calculation were performed with ImageJ 1.54 software (National Institutes of Health (NIH), Bethesda, MD, USA).

### 4.3. Biological Samples and Preparation

Biological samples, consisting of follicular fluid and blood serum, were obtained from Holstein-Friesian cows (n = 21; 12 to 30 months old) participating in a commercial ovum pick-up (OPU) program for IVEP. The collection was performed as part of a standard veterinary procedure. Follicular fluid was recovered during routine ultrasound-guided transvaginal aspiration of ovarian follicles. Immediately following collection, the follicular fluid was centrifuged at 3000× *g* for 10 min at 4 °C to pellet cellular debris. Alongside, whole blood was collected via jugular venipuncture into tubes containing a clot activator. The blood was allowed to clot for 30 min at room temperature and subsequently centrifuged under identical conditions to isolate the serum. The resulting supernatants (serum and cell-free follicular fluid) were aliquoted into sterile cryovials, stored in the vapor phase of liquid nitrogen (range between −150 °C to −196 °C) until analysis. As the samples were collected during routine, non-experimental veterinary procedures established in commercial practice, no formal ethical approval was required under the Polish Act of 15 January 2015 on the Protection of Animals Used for Scientific or Educational Purposes, which implements the EU Directive 2010/63/EU.

### 4.4. Biosensor Preparation Protocol

The preparation of the biosensor was a multi-stage process designed to create a stable, specific, and reproducible sensing interface. The initial step was the formation of a self-assembled monolayer (SAM), which serves as a foundational linker layer. The gold sensor chips were meticulously cleaned to ensure a pristine surface before being immersed in a 20 mM ethanolic solution of cysteamine. During an extended incubation of at least 12 h at room temperature, the thiol groups of the cysteamine molecules spontaneously formed strong, covalent bonds with the gold surface, leading to the creation of a dense and well-ordered SAM. This process resulted in a uniformly functionalized surface with outwardly oriented terminal amine groups, which provide reactive sites for the subsequent covalent attachment of the biorecognition element. Following incubation, the chips were thoroughly rinsed with absolute ethanol and deionized water to remove cysteamine molecules nonspecifically adsorbed to the surface.

The second stage, antibody immobilization, involved the covalent attachment of the specific biorecognition element to the prepared surface using standard carbodiimide chemistry. A freshly prepared mixture of EDC and NHS in carbonate buffer was used to activate the carboxyl groups on the anti-IL-10 polyclonal antibody, converting them into highly reactive, semi-stable NHS-esters. A 3 µL aliquot of this activated antibody solution was then precisely spotted onto each of the nine amine-functionalized measurement fields. During a 1 h incubation at 37 °C, the NHS-esters reacted with the primary amines of the cysteamine layer to form stable, covalent amide bonds. This procedure ensured a robust attachment of the antibody to the sensor surface, which is crucial for its subsequent antigen-binding activity.

Following immobilization, a washing and blocking step was performed to minimize non-specific signals and ensure assay specificity. The chip was first washed with HBS-ES buffer and deionized water to remove any physically adsorbed or weakly bound antibody molecules. To prevent non-specific adsorption of proteins from the sample matrix during analysis, any remaining active sites on the sensor surface were passivated. This was accomplished by incubating the chip with a solution of bovine serum albumin (BSA, 1 mg/mL), a common blocking agent that adsorbs unoccupied spaces. A final rinsing step removed excess BSA, leaving a specific and bioinert surface ready for analysis.

The final step was analyte measurement. A 3 μL volume of the analytical sample (either a calibration standard or a biological sample) was applied to each measurement field. For biological samples, follicular fluid was diluted twofold with PBS, whereas serum was analyzed without dilution. The samples were subsequently incubated for 10 min to allow the immobilized antibodies to capture the IL-10 analyte. An incubation period of 10 min had been previously optimized to yield a significant binding signal while keeping the overall assay time short. Following incubation, the surface was gently rinsed with HBS-ES buffer and water to remove the sample matrix and unbound molecules, ensuring the final measured signal reflected only specifically bound analyte.

Following the completion of an analytical run, the chips were subjected to a regeneration protocol to prepare the surface for a new functionalization cycle, based on a previously described procedure [14]. This was achieved by rinsing the used biosensors extensively with a mixture containing 100 mM NaOH and 1% Triton X-100. Subsequently, the chips were thoroughly washed with water and dried under a stream of high-purity argon. This procedure facilitates the alkaline hydrolysis of the amide bond between the antibody and the cysteamine linker, effectively stripping the biological layers while leaving the underlying thiol monolayer intact. This regeneration process allows for the reuse of a single gold chip for up to five analytical cycles.

### 4.5. SPRi Measurement Procedure

The prepared sensor chip was mounted on a prism in the SPRi device. First, a baseline image of the antibody-functionalized surface was recorded. After the 10-min incubation with the IL-10-containing sample and subsequent washing, a second image was captured. The binding of the analyte to the immobilized antibodies causes a change in the local refractive index at the sensor surface, which is detected as a change in the intensity of the reflected light. The final SPRi signal, expressed in arbitrary units (AU), was calculated as the difference in signal intensity before and after the interaction.

### 4.6. Method Validation

The analytical method was comprehensively validated according to established bioanalytical guidelines to ensure its reliability, accuracy, and robustness. The validation process encompassed the assessment of several key performance characteristics. Initially, the optimization of experimental conditions was performed to achieve the highest possible signal-to-noise ratio, which involved systematically varying the SPR angle of incidence and the concentration of the immobilized antibody to identify parameters yielding the maximum specific signal. Subsequently, a calibration curve was established to define the relationship between analyte concentration and instrument response by analyzing seven IL-10 standards of known concentrations. The method’s sensitivity was then determined by calculating the limit of detection (LOD) and limit of quantification (LOQ) from the calibration data, defined as 3.3 σ/S and 10 σ/S, respectively (where σ is the standard deviation of the blank and S is the slope).

Following the initial characterization, the method’s performance was further evaluated. Precision and accuracy were thoroughly assessed to determine reproducibility and correctness through intra-assay (repeatability) and inter-assay (intermediate precision) analyses at four concentration levels. The selectivity of the biosensor was confirmed by testing for cross-reactivity against high concentrations of structurally related cytokines (IL-1β, IL-6, and IFN-γ) to ensure specificity. The short-term stability and consistency of the measurement were confirmed by assessing repeatability through six consecutive measurements of both a standard solution and a real biological sample. To benchmark the performance of the developed biosensor against established commercial methods, a comparative analysis was included as part of the validation. The same panel of biological samples was also tested using two commercial enzyme-linked immunosorbent assay (ELISA) kits: the Bovine Interleukin-10 (IL-10) ELISA Kit (MyBioSource, San Diego, CA, USA; cat. no. MBS8726) and the Bovine Interleukin-10 (IL-10) ELISA Kit (Cusabio, Houston, TX, USA; cat. no. CSB-E06754Bo). The assays were performed according to the manufacturer’s instructions. To confirm the presence of IL-10 in the samples, a dot blot assay was performed using a rabbit polyclonal antibody specific for bovine IL-10 (MyBioSource, San Diego, CA, USA).

## 5. Conclusions

This study developed and validated an SPRi biosensor for the quantification of bovine IL-10, demonstrating its performance in native serum and individual follicular fluid. The platform achieved a diagnostic range of 1–1000 pg/mL with an LOQ of 1.49 pg/mL, high selectivity against related cytokines, and acceptable precision and accuracy. The 3 × 3 array enabled nine simultaneous determinations using approximately 3 µL per spot in about 30 min and allowed chip regeneration, reducing sample demand and hands-on time compared to conventional immunoassays. In matched samples, two commercial ELISAs were below their stated LOQs, whereas SPRi returned quantitative values, confirming practical advantage in bovine matrices. IL-10 concentrations were higher in follicular fluid than in serum, indicating compartmentalization and supporting local measurement for reproductive-tract immunity. These results establish a first-in-species SPRi capability for bovine IL-10 that is suitable for scarce matrices, providing a foundation for standardized measurements and prospective studies linking follicular-fluid IL-10 to IVEP outcomes alongside cross-platform calibration across key bovine matrices.

## Figures and Tables

**Table 1:** Analytical specificity of the biosensor against interfering cytokines.

Interferent Concentration ^1^	SPRi Signal	C_IL-10_ [pg/mL]	Note
500 pg/mL	1418.541	0.756	<LOQ
	1417.639	0.605	<LOQ
	1417.572	0.594	<LOQ
2000 pg/mL	1416.75	0.458	<LOQ
	1418.798	0.798	<LOQ
	1417.866	0.643	<LOQ

^1^ Mixture of IL-6, IL-1β, and IFN-γ; C_IL-10_—IL-10 concentration.

**Table 2:** IL-10 concentrations in bovine serum and follicular fluid determined by SPRi biosensor.

Sample ^1^	SPRi Signal [AU]	Dilution	C_IL-10_ [pg/mL]	Body Fluid
1	4252.8635	2	328.62	follicular fluid
2	7815.8005	2	738.46	follicular fluid
3	8443.117	2	810.62	follicular fluid
4	5348.612	2	454.66	follicular fluid
5	7521.1405	2	704.57	follicular fluid
6	7826.7345	2	739.72	follicular fluid
7	4185.5195	2	320.87	follicular fluid
8	5597.174	2	483.25	follicular fluid
9	7168.9685	2	664.06	follicular fluid
A	5018.086	1	208.32	serum
B	1816.113	1	24.16	serum
C	3811.059	1	138.90	serum
D	4448.985	1	175.59	serum
E	1519.105	1	7.08	serum
F	7277.147	1	338.25	serum
G	6977.786	1	321.03	serum
H	4709.417	1	190.57	serum
I	4199.57	1	161.25	serum

^1^ Samples 1–9 were obtained from follicular fluid by ovum pick-up (OPU); samples A–I were obtained from serum; C_IL-10_: IL-10 concentration. SPRi signal [AU] denotes arbitrary units calculated as the difference between baseline and post-incubation reflected intensity after the 10-min binding step. Corresponding ELISA measurements on the same samples (MyBioSource, cat. no. MBS8726; Cusabio, cat. no. CSB-E06754Bo) were below the manufacturers’ stated LOD/LOQ, were classified as non-quantifiable.

## Data Availability

The original contributions presented in this study are included in the article. Further inquiries can be directed to the corresponding authors.
